# *Plasmodium vivax* malaria relapses at a travel medicine centre in Rio de Janeiro, a non-endemic area in Brazil

**DOI:** 10.1186/1475-2875-11-245

**Published:** 2012-07-28

**Authors:** Renata S Pedro, Lusiele Guaraldo, Dayse P Campos, Anielle P Costa, Cláudio T Daniel-Ribeiro, Patrícia Brasil

**Affiliations:** 1 Instituto de Pesquisa Clínica Evandro Chagas (IPEC), Fundação Oswaldo Cruz (Fiocruz), Rio de Janeiro. Av. Brasil 4365. Manguinhos, Rio de Janeiro, RJ - CEP 21.045-900, RJ –, Brazil; 2 Centro de Pesquisa, Diagnóstico e Treinamento em Malária (CPD-Mal), Fiocruz and Secretaria de Vigilância em Saúde (SVS), Ministério da Saúde (MS), Brazil; 3 Laboratório de Pesquisas em Malária. Instituto Oswaldo Cruz, Fiocruz. Pavilhão Leônidas Deane, 5° andar. Av. Brasil 4365. Manguinhos, Rio de Janeiro, RJ, CEP 21.045-900, RJ, Brazil

**Keywords:** *Plasmodium vivax* malaria, Relapse, Therapeutic failure, Weight-based dosing

## Abstract

**Background:**

Malaria is a potentially severe disease widely distributed in tropical and subtropical regions worldwide. Clinically, the progression of the disease can be life-threatening if it is not promptly diagnosed and properly treated. Through treatment, the radical cure of *Plasmodium vivax* infection can be achieved, thus preventing potential relapses and the emergence of new cases outside the Amazon region in Brazil. Surveillance for therapeutic failure in non-endemic areas is advantageous, as it is unlikely that recurrence of the disease can be attributed to a new malaria infection in these regions.

**Methods:**

An observational study of 53 cases of *P. vivax* and mixed (*P. vivax* and *Plasmodium falciparum*) malaria was conducted at a travel medicine centre between 2005 and 2011 in Rio de Janeiro and a descriptive analysis of the potential factors related to recurrence of *P. vivax* malaria was performed. Groups with different therapeutic responses were compared using survival analysis based on the length of time to recurrence and a set of independent variables thought to be associated with recurrence.

**Results:**

Twenty-one relapses (39.6%) of *P. vivax* malaria were observed. The overall median time to relapse, obtained by the Kaplan-Meier method, was 108 days, and the survival analysis demonstrated an association between non-weight-adjusted primaquine dosing and the occurrence of relapse (p < 0.03). Primaquine total dose at 3.6 mg/kg gave improved results in preventing relapses.

**Conclusions:**

A known challenge to individual cure and environmental control of malaria is the possibility of an inappropriate, non-weight-based primaquine dosing, which should be considered a potential cause of *P. vivax* malaria relapse. Indeed, the total dose of primaquine associated with non-occurrence of relapses was higher than recommended by Brazilian guidelines.

## Background

Malaria is a potentially severe disease widely distributed in tropical and subtropical regions worldwide. Because of present-day ease of travel and the magnitude of migratory movements, it can be considered a global problem. Death can occur in non-immune individuals if the disease is not diagnosed in time and treated appropriately. Most malaria cases in the Americas occur in Brazil, where *Plasmodium vivax* is responsible for 84% of the cases registered, 99.8% of which occur in the Brazilian Amazon [1-3].

Relapse, a common feature of this type of malaria, is defined as the reappearance of the disease and parasitaemia after initial eradication of blood forms. It is caused by the survival of hypnozoites (dormant forms of *P. vivax* or *Plasmodium ovale* latent in the liver) [4]. Although *P. vivax* malaria is known for its benign course, there are reports of more complicated cases in the Brazilian Amazon [5,6] and elsewhere [7,8].

Failure in deactivating the hepatic hypnozoite forms of *P. vivax* by treatment can be due to several factors. Although other factors – such as individual pharmacokinetic variations (poor absorption, rapid elimination or low biotransformation of drugs), adherence to the treatment, drug interactions, adverse drug events, dose adjustment to body weight, and treatment duration – are directly related to the infection’s response to anti-malarial treatment, primaquine tolerance is a very important issue that few studies have addressed [9,10].

Detection of treatment failure or relapse may prevent malaria’s introduction into unaffected areas where the vector remains a regional menace. As there is no standardized *in vitro* method for assessing the hypnozoiticidal effects of treatment, patient follow-up – particularly outside the transmission areas – remains the best way to monitor the susceptibility of *P. vivax* to primaquine.

This paper aims to describe the frequency of *P. vivax* malaria relapse at a sentinel unit in a non-Amazonian region and the factors potentially associated with this event.

## Methods

### Study design, location, and patient selection

This descriptive study was conducted from January 2005 to October 2011 at a specialized post-travel care clinic for adolescents and adults (≥12 years old) at the Instituto de Pesquisa Clínica Evandro Chagas (IPEC) in Rio de Janeiro, a state located outside the Amazon region and where malaria transmission does not occur.

Eighty-nine patients treated for vivax malaria during the period were studied. Those who had returned to an area of malaria transmission after starting treatment or who had less than 28 days of follow-up were excluded. Accordingly, the analysis contemplated 53 patients (59.6%). These were followed up for 28 to 408 days (median = 62 days).

Of the 53 patients studied, 47 (88.7%) were infected only with P. *vivax*, and the other six (11.3%), with *P. vivax* and *Plasmodium falciparum* (mixed malaria). Males predominated (75.5%), and ages ranged from 14 to 63 years (median = 29 years). Most of the travellers came from South America: Brazilian Amazon (71.6%), French Guiana (13.2%), Venezuela (3.8%), Guyana (1.9%) and Suriname (1.9%); the rest were from Angola (3.8%) and Indonesia (3.8%).

*Plasmodium vivax* infections were treated with chloroquine (600 mg on the first day, 450 mg on the second and third days) and primaquine. Mixed infections caused by *P. falciparum* and *P. vivax* were treated with mefloquine (20 mg/kg body weight, single dose) and primaquine, a therapeutic regimen still used by the Brazilian Malaria Therapy Guidelines until 2009, or with artesunate + mefloquine (200 mg + 400 mg for three days) and primaquine [11,12]. Primaquine was prescribed according to the Brazilian Malaria Therapy Guidelines current at the time of the malaria diagnosis and treatment: (1) total dose of 3.5 mg/kg or fixed total dose of 210 mg regardless of weight, following the 2001 guidelines [11]; or (2) dose adjusted to stratified patient weight, with total dose ranging from minimal 3.0 to maximal 3.4 mg/kg, following the 2009 guidelines [12]. Over the entire study period, during which both guidelines were used, the total dose of primaquine ranged from 2.2 to 4.9 mg/kg. Primaquine total dose was adjusted in 69.8% of treatments. Treatment duration varied from 7 to 13 days (short regimen) or from 14 to 28 days (long regimen). A primaquine short regimen was prescribed in most of the treatments (66.0%) and the remaining patients were treated with primaquine long regimen. The choice of primaquine prescription regimen was made at doctors’ discretion at the outpatient clinic. All medicines were prescribed for home use, patients were educated on the importance of following the prescribed regimen exactly and drug administration was not supervised.

The project was approved by the IPEC Research Ethics Committee, Fiocruz (No. 0031.0.009.000-10), and the information obtained was kept strictly secret and confidential.

### Detection and quantification of malaria parasites

Thin and thick blood smears were stained with Giemsa and analysed by light microscopy using an immersion oil lens (x100 objective magnification) to identify the parasite species and determine the density of *Plasmodium* asexual and sexual stages, according to standard procedures [13]. Malaria smears were examined for diagnosis at each follow-up visit until parasitological clearance and at days 7, 14, 21, 28, 40, and 60 of treatment and at any time in case of fever recurrence.

### Data collection

Data were collected from the clinic and IPEC pharmacovigilance databases. The variables considered were (a) therapeutic response, classified as relapse or non-relapse; (b) gender; (c) age; (d) adverse drug events; (e) primaquine treatment duration; (f) whether or not the primaquine dose was weight-adjusted; (g) and primaquine total dose. Relapse was considered as the recurrence of parasitaemia following parasitological remission after 28 days from starting treatment for vivax malaria. Primaquine treatment duration was considered an indirect factor related to adherence to treatment, since total dose was the same, regardless of regimen.

### Statistical analysis

Descriptive analysis was performed on factors potentially related to relapse of *P. vivax* malaria. Groups with different therapeutic responses were compared using survival analysis. Survival time was defined as the number of days elapsed between starting treatment and disease recurrence. The event of interest was relapse of vivax malaria*.* Cases with no evidence of relapse during the study period were censored at the last day of follow-up. The Kaplan-Meier method was applied to calculate the survival functions, which are defined as the probability of relapse not occurring. The log-rank test was used to compare survival functions among categories of each variable. First the hazard ratios (HR) with 95% confidence intervals were calculated, and then the Cox proportional hazards model. Analysis of Schoenfeld and Martingale residuals was used to assess the Cox method assumptions for covariates of the adjusted model. The smoothing function (spline) was applied for the non-linear continuous variable “primaquine total dose” in order to show the effect of dose on relapses graphically. Spline coefficient values of less than zero indicate occurrence of protection against relapse. The significance level for all hypothesis testing was set at p ≤ 0.05. Data were analysed using R software, version 2.14.2.

## Results

**Figure 1:**
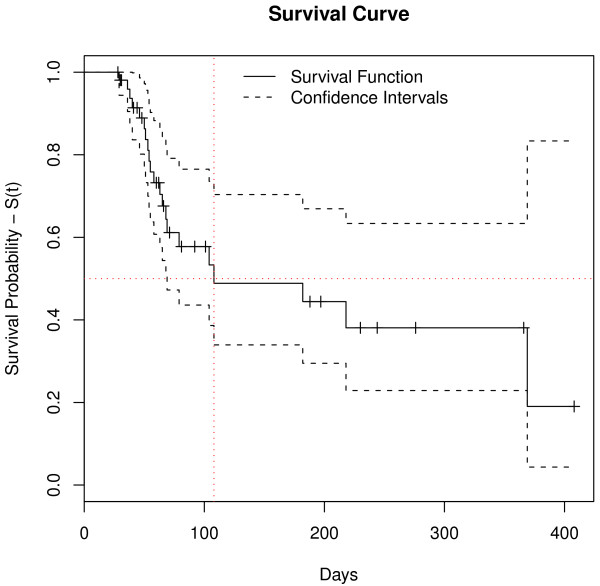
**Survival curve.** Overall Kaplan-Meier survival curve for *P. vivax* malaria relapse among patients included in the study (IPEC, 2005-2011). Survival function is defined as the probability of relapse not occurring.

**Figure 2:**
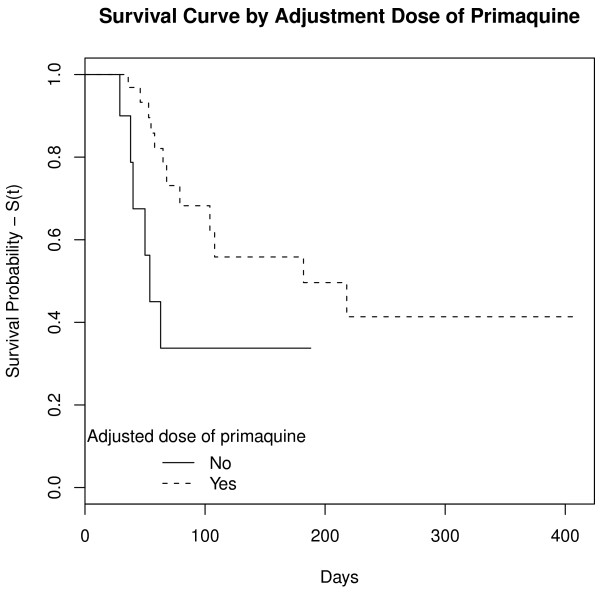
**Survival curve by adjusted dose of primaquine.** Patients with adjusted doses of primaquine had a higher survival and, consequently, lower relapse rate (p < 0.03) than those with doses not adjusted for patient body weight. IPEC, 2005-2011.

**Figure 3:**
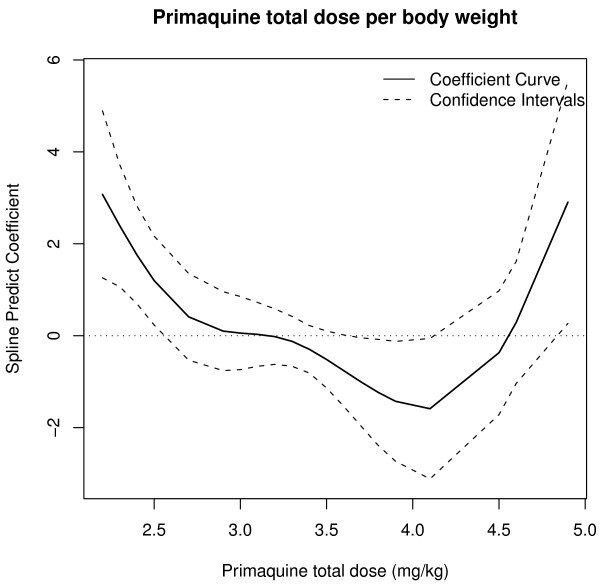
**Smoothing function (spline) for primaquine total dose by weight.** Primaquine total dose ranging from 3.6 to 4.1 mg/kg seems to offer protection against relapse.

Twenty-one relapses (39.6%) of *P. vivax* malaria were observed. Most (n = 14) occurred in patients from the Brazilian Amazon; the other patients were from French Guiana (n = 4), Venezuela (n = 2) and Indonesia (n = 1). These relapses occurred in 45.7% of patients treated with primaquine short regimen (N = 35) and in 28.6% of patients treated with primaquine long regimen (N = 14) (p > 0.05). Thirty-two cases were censored in survival analysis, because relapse did not occur during the study period (Table 1). All patients with relapse were re-treated. In one case, poor quality primaquine was considered a potential cause of relapse, after more than three relapses occurred in a short period of time. However, quality analysis (physical appearance, content and dissolution) of the chloroquine and primaquine tablets revealed no evidence of poor quality. Time to relapse ranged from 29 to 369 days after start of treatment. In survival analysis, overall median time to relapse obtained by the Kaplan-Meier method was 108.0 days [95% CI 69 – not achieved], i.e., 50% of relapses occurred less than 108 days after start of treatment (Figure 1). Patients with adjusted doses of primaquine had higher survival and, consequently, lower relapse rate (p < 0.03) than those with no body weight-based dosing adjustments (Figure 2). The other covariates analysed were not statistically significant (Table 1). The risk of relapse, calculated by the Cox proportional hazards model, was 2.94 times greater [95% CI 1.09 - 8.33] for patients with no dosing adjustments than for patients with dosing adjustments; and, after adjustment for covariables (gender, age and primaquine treatment duration), estimated risk of relapse were 3.13 [95% CI 1.08-9.09] (Table 2). Primaquine total dose of 3.6 mg/kg gave superior results in preventing relapses (protection factor). The probability of relapse not occurring, when primaquine total dose ranged from 3.6 to 4.1 mg/kg, is shown graphically (Figure 3). The increased risk of relapse with primaquine total dose lower than 3.6 mg/kg in relation to doses ranging from 3.6 to 4.1 mg/kg was statistically significant. The estimated risk adjusted for covariables was 9.36 [1.06-82.23]. Both Cox proportional hazards models returned strong concordance, with values higher than 70% (Table 2).

**Table 1:** **Patients with*P. vivax*or mixed (*P. vivax*and*P. falciparum*) malaria treated at IPEC (2005-2011), classified according to therapeutic response**

	**Non-Relapse**		**Relapse**		**Median [CI 95%] (days)**	**p (Log-Rank)**
	**n**	**%**	**N**	**%**		
**Sample**	32	100	21	100	108 [69-NA}	
**Gender**						
Men	22	68.8	18	85.7	104 [68-NA]	0.16
Women	10	31.2	3	14.3	NA [58-NA]	
**Age range (years)**						
14-30	14	43.8	14	66.7	79 [58-NA]	0.22
31-50	13	40.6	4	19.0	369 [108-NA]	
≥51	5	15.6	3	14.3	68 [51-NA]	
Adverse drugs events (ADE)*						
Yes	10	31.3	2	9.5	NA [58-NA]	0.20
No	17	53.1	12	57.1	108 [68-NA]	
Duration of primaquine therapy*						
Short regimen	19	59.4	16	76.2	79 [65-NA]	0.34
Long regimen	10	31.3	4	19.0	218 [104-NA]	
Primaquine total dose adjusted by weight*						
Yes (≥ 3.2 mg/kg)	25	78.1	12	57.1	182 [104-NA]	0.03
No (< 3.2 mg/kg)	5	15.6	6	28.3	54 [40-NA]	

* Missing data was 22.6%, 7.5% and 9.4% for ADE, duration of treatment with primaquine and primaquine dose adjusted by weight, respectively.

NA = not achieved.

**Table 2:** **Crude and adjusted hazard ratio (HRs) for potential factors related to relapse of*P. vivax*malaria**

	**Crude (n = 53)**		Adjusted* (n = 48)			
			**Model 1**		**Model 2**	
	**HR [CI 95%]**	**p (Wald)**	**HR [CI 95%]**	**p (Wald)**	**HR [CI 95%]**	**p (Wald)**
**Gender**						
Men	2.36 [0.69–8.07]	0.17	2.23 [0.63-7.84]	0.21	3.9 [0.59-26.81]	0.16
Women	1		1		1	
**Age (years)** **	0.97 [0.93-1.02]	0.25	0.95 [0.90-1.01]	0.09	0.94 [0.89-0.99]	0.03
**Adverse drug events (ADE)**						
Yes	0.39 [0.09-1.74]	0.22	-	-	-	-
No	1		-	-	-	-
**Duration of primaquine therapy**						
Short regimen	1	0.18	1	0.14	1	0.04
Long regimen	0.46 [0.15-1.42]		0.42 [0.13-1.33]		0.28 [0.08-0.98]	
**Primaquine total dose adjusted by weight**						
Yes (≥ 3.2 mg/kg)	1	0.03	1	0.04	-	-
No (< 3.2 mg/kg)	2.94 [1.09-8.33]		3.13 [1.08-9.09]		-	-
**Primaquine total dose (mg/kg)**						
**< 3.6**	5.24 [0.68-40.15]	0.24	-	-	9.36 [1.06-82.23]	0.04
**3.6 – 4.1**	1		-	-	1	
**> 4.1**	7.09 [0.64-78.74]		-	-	15.4 [0.79-300.57]	0.07
**Concordance**	-	-	0.73		0.75	
**R square**	-	-	0.23		0.28	
**p (Wald)**	-	-	0.06		0.08	

* Cox regression models. **Continuous variable.

## Discussion

The current study, performed in the city of Rio de Janeiro, where malaria transmission does not occur, showed that approximately 40% of patients treated within the last six years had relapses of *P. vivax* malaria and that failure to adjust primaquine dosing was the most important factor in the occurrence of relapse. Still, the primaquine total dose related to non-occurrence of relapse was higher than recommended by the Brazilian guidelines. Relapse is considered to be the parasitological recurrence of *P. vivax* after 28 days of treatment in patients who do not return to an endemic area [4]. Non-endemic areas, therefore, offer advantages for monitoring therapeutic failures due to improper treatment or drug resistance since risk of re-infection is minimal or absent.

A retrospective study in São Paulo, Brazil [14] among 1,347 cases of *P. vivax* malaria showed a relapse rate of 25% over seven years. However, Duarte *et al*[15] in Cuiaba, Brazil and Kitchener *et al*[16] in Melbourne, Australia, prospectively followed patients for two years and showed relapse rates of 14% in 50 patients and 24% in 318 patients, respectively. The higher frequency recorded (40%) could be explained by the smaller sample in this study or by the fact that data for Brazil obtained in 2005-2011 were compared to data obtained in 1991 and in 2003, and concern with primaquine tolerance may have been different in these two periods. The data reported by Kitchener in Australia certainly refer to patients who acquired malaria in quite different localities, and the situations may not be comparable to data obtained in Brazil.

Relapses can occur months or even years after the time of initial infection, with a maximum recorded time of four years [17-20]. In the present study, 86% of relapses occurred up to day 180 after start of treatment, in agreement with the study by Boulos [14], where 95% of relapses took place before day 180, and the longest time to relapse (369 days) occurred in a patient with a *P. vivax* infection acquired on a trip to Indonesia, where the prevalence of primaquine-tolerant *P. vivax* is higher than elsewhere [21]. Although there is no standardized or validated method for diagnosing resistance to primaquine [9], the demonstration of primaquine-tolerant *P. vivax* in the South Pacific, Southeast Asia, Western Pacific, Oceania and Central America [9,10,21,22], associated with travellers’ high mobility, should sound an alert to the possibility of tolerance being introduced into the Brazilian Amazon [23], from where most relapse cases came.

Besides drug resistance, other factors, such as adherence to the treatment, treatment duration, quality of anti-malarials, adverse drug events and dose adjustment to body weight may be related to the infection’s response to anti-malarial treatment.

Adherence to anti-malarial treatment remains an important key to malaria control [24,25]. Direct methods (drug serum levels and supervised treatment), and indirect methods (interviews) can quantify adherence to treatment. Although supervision is directly related to treatment effectiveness, health education generally tends to approximate the efficacy of non-supervised and supervised treatments [26,27]. Patients at IPEC are educated on the importance of adherence to regular therapy. This procedure resulted in high adherence behaviour, as previously demonstrated [28] by using Morisky’s assessment protocol [29].

Anti-malarials’ quality ensures their therapeutic effectiveness in eliminating parasites from the blood and achieving malaria treatment success. Studies performed in Brazil indicate that poor quality anti-malarial drugs and inappropriate storage conditions may contribute to the development of parasite resistance [30,31]. In the present study, poor quality primaquine was suspected as a potential cause of relapse after four relapses in a short time, three of them in one patient, after treatment with the same batch of primaquine [32]. Impaired drug quality was discounted, however, and the patient was successfully treated after adjusting the dose of primaquine.

Most adverse drug reactions were mild and involved disorders of the gastrointestinal system – nausea, vomiting and diarrhoea, which could theoretically have reduced drug absorption. However, in the applied model, relapses were not associated with the occurrence of adverse drug reactions in the study.

Primaquine is currently the only drug available for treatment of the latent liver forms of the parasite. The literature suggests that a 14-day course of primaquine may be more effective than a 7-day course, and this superiority may be attributable to significant accumulation of the drug’s active metabolite after longer administration [33-35]. On the other hand, more prolonged treatment may hinder patient adherence, as clinical cure usually occurs in less than 72 hours [28,36]. It seems, however, that this did not occur in the current study, since the proportion of relapses after long-regimen treatment was lower than after the short regimen (p > 0.05) and patient adherence rates were high [28].

Conversely, the results clearly indicate an association between non-weight-based primaquine dosing and the occurrence of relapses (p < 0.03). These results agree with studies that suggested dosing adjustments for patients weighting over 70 kg at a time when obesity was not yet a global problem [37-40].

Although it is already known that weight-based dosing of primaquine is required to prevent relapses, it is important that an appropriate weight-based primaquine total dose be determined and confirmed to guide clinical decisions. In this study, a total dose of 3.2 mg/kg indicates protection against relapses. However, the probability of relapse occurring still exists given the upper limit of the confidence interval. Therefore, in order to treat the patients in this study more effectively, a more appropriate dose would be higher (between 3.6 and 4.1 mg/kg) than the one recommended by the Brazilian Ministry Health (from 3.0 to 3.4 mg/kg [11,12]). The more effective dose range demonstrated here is also closer to the World Health Organization’s recommendation of 3.5 mg/kg in 14 days for radical cure of vivax malaria [41]. This dose does not apply to individuals infected in countries from Oceania and Southeast Asia, where the total recommended dose is 6.0 - 7.0 mg/kg in 14 days [42-44]. The increased risk of relapse foreseen by the smoothing function (spline) above 4.1 mg/kg primaquine total dose by weight, as shown in Figure 3, was not statistically significant.

Although small sample size may be considered a limitation on the statistical analysis, an association was found between adjusted primaquine dose and protection against relapse. Also, follow-up of infected patients in non-endemic areas is a good methodological approach to define a true relapse, because no confounding re-infection is possible in such areas.

The results reported here corroborate the conclusion of Fernando *et al*[45] that there is enough evidence to support the use of higher total doses of primaquine to prevent relapses in weight-adjusted treatment.

## Conclusions

The study suggests that the total dose of primaquine above which relapses do not occur is 3.6 mg/kg. This conclusion is in agreement with WHO and other international guidelines, but is higher than the total dose recommended by Brazilian Ministry of Health Guidelines. Given the ease of travel, the evidence of growing primaquine tolerance around the world and the possibility of its introduction into the Amazon basin, it would be advisable to review the recommendations of the Brazilian Malaria Control Programme. Finally, it may be argued that malaria control programmes should use public health educational messaging campaigns to alert travel clinicians to the fact that risk of therapeutic failure can be reduced by tailoring drug therapies individually to each patient.

## Competing interests

The authors have no conflicts of interest to disclose.

## Authors' contributions

RSP: responsible for conception and design of the work, data collection, data analysis, data interpretation and drafting the manuscript. PB: responsible for conception and design of the work, data analysis, data interpretation, literature review and reviewing the manuscript. LG: responsible for conception and design of the work, data analysis, data interpretation, literature review and reviewing the manuscript. DC: responsible for data analysis and interpretation. APC: responsible for data collection and helped to review the text. CTDR: helped in the design of the work and reviewed the text up to the final version to be published. All authors read and approved the final manuscript.

## References

[B1] Oliveira-Ferreira J Lacerda MVG Brasil P Ladislau JLB Tauil PL Daniel-Ribeiro CT Malaria in Brazil: an overview Malar J 2010 9 115 10.1186/1475-2875-9-115 20433744 PMC2891813

[B2] Daniel-Ribeiro CT Lacerda MGV Oliveira-Ferreira J Paludisme dû à Plasmodium vivax en Amazonie brésilienne: quelques aspects de son épidémiologie, de ses manifestations cliniques et des réactions immunitaires naturellement acquises Bull Soc Path Exot 2008 101 243 248 18683322

[B3] Brasil, Ministério da Saúde Situação epidemiológica da malária no Brasil, ano de 2007 2008 Ministério da Saúde, Brasília

[B4] Baird JK Leksana B Masbar S Fryauff DJ Sutanihardja MA Suradi F Wignall FS Hoffman L Diagnosis of resistance to chloroquine by Plasmodium vivax: timing of recurrence and whole blood chloroquine levels AmJTrop Med Hyg 1997 56 Suppl 6 621 626 10.4269/ajtmh.1997.56.621 9230792

[B5] Costa AP Bressan CS Pedro RS Valls-de-Souza R Silva S Souza PR Guaraldo L Ferreira-da-Cruz MF Daniel-Ribeiro CT Brasil P Diagnóstico tardio da malária em área endêmica de dengue na extra-Amazônia Brasileira: experiência recente de uma unidade sentinela no estado do Rio de Janeiro Rev Soc Bras Med Trop 2010 43 571 574 10.1590/S0037-86822010000500020 21085872

[B6] Lacerda MVG Manifestações clínicas e patogênses da plaquetopenia na malária. PhD Thesis 2007 Universidade de Brasília, Brasília

[B7] Joon Young S Cheong Won P You Mee J Jeong Yun K Jeong Hyun K Hyo Joong Y Chi Hoon K Chae Seung L Hee Jin C Woo Joo K Two cases of Plasmodium vivax malaria with the clinical picture resembling toxic shock AmJTrop Med Hyg 2007 77 609 611 17978057

[B8] Karyana M Burdarm L Yeung S Kenangalem E Wariker N Maristela R Umana KG Vemuri R Okoseray MJ Penttinen PM Ebsworth P Sugiarto P Anstey NM Tjitra E Price RN Malaria morbidity in Papua Indonesia, an area with multidrug resistant Plasmodium vivax and Plasmodium falciparum Malar J 2008 7 148 10.1186/1475-2875-7-148 18673572 PMC2518158

[B9] Baird JK Resistance to therapies for infection by Plasmodium vivax Clin Microbiol Rev 2009 22 508 534 10.1128/CMR.00008-09 19597012 PMC2708388

[B10] Signorini L Matteelli A Castelnuovo F Castelli F Oladeji O Carosi G Short report: primaquine-tolerant Plasmodium vivax in an Italian traveler from Guatemala AmJTrop Med Hyg 1996 55 472 473 10.4269/ajtmh.1996.55.472 8940974

[B11] Brasil, Ministério da Saúde Manual de Terapêutica da Malária 2001 Ministério da Saúde, Brasília

[B12] Brasil, Ministério da Saúde Guia Prático de Tratamento da Malária no Brasil 2009 Ministério da Saúde, Brasília

[B13] Brasil MS Manual de diagnóstico laboratorial da malária 2005 Ministério da Saúde, Brasília 112

[B14] Boulos M Amato Neto V Dutra AP Di Santi SM Shiroma M Análise da freqüência de recaídas de malária por Plasmodium vivax em região não endêmica Rev Inst Med Trop Sao Paulo 1991 33 143 146 10.1590/S0036-46651991000200009 1844384

[B15] Duarte EC Pang LW Ribeiro LC Fontes CJF Association of subtherapeutic dosages of a standard drus regimen with failures in preventing relapses of vivax malaria AmJTrop Med Hyg 2001 65 471 476 10.4269/ajtmh.2001.65.471 11716100

[B16] Kitchener S Nasveld P Bennett S Torresi J Adequate primaquine for vivax malaria J Travel Med 2005 12 133 135 15996441 10.2310/7060.2005.12306

[B17] Warrell DA Warrell DA, Gilles HM Clinical features of malaria Essential Malariology 2002 4 Oxford University Press, New York 192

[B18] Fairhurst RM Wellems TE Mandell GL, Bennett JE, Dolin R Plasmodium species (Malaria) Mandell, Douglas and Bennett's Principles and Practice of Infectious Diseases 2009 7 Churchill Livingstone, New York 3437 3462

[B19] Mangoni ED Severini C Menegon M Romi R Ruggiero G Majori G Case Report: an unusual late relapse of Plasmodium vivax malaria AmJTrop Med Hyg 2003 68 159 160 12641405

[B20] White NJ Determinants of relapse periodicity in Plasmodium vivax malaria Malar J 2011 10 297 10.1186/1475-2875-10-297 21989376 PMC3228849

[B21] Schwartz IK Lackritz EM Chloroquine resistant Plasmodium vivax from Indonesia N Engl J Med 1991 324 927 2000121 10.1056/NEJM199103283241317

[B22] Rieckmann H Davis DR Hutton DC Plasmodium vivax resistance to chloroquine? Lancet 1989 334 1183 1184 10.1016/S0140-6736(89)91792-3 2572903

[B23] Ananias LC Escalante A Imwong M Snounou G The genetic diversity of Plasmodium vivax populations Trends Parasitol 2003 19 220 226 10.1016/S1471-4922(03)00085-0 12763428

[B24] Duarte EC Gyorkos TW Self-reported compliance with last malaria treatment and occurrence of malaria during follow-up in a Brazilian Amazon population Trop Med Int Health 2003 8 518 524 10.1046/j.1365-3156.2003.01042.x 12791057

[B25] Yeung S White NJ How do patients use antimalarial drugs? A review of the evidence Trop Med Int Helath 2005 10 121 138 10.1111/j.1365-3156.2004.01364.x 15679555

[B26] Leslie T Rab MA Ahmadzai H Durrani N Fayaz M Kolaczinski J Rowland M Compliance with 14-day primaquine therapy for radical cure of vivax malaria - a randomized placebo-controlled trial comparing unsupervised with supervised treatment Trans R Soc Trop Med Hyg 2004 98 168 173 10.1016/S0035-9203(03)00041-5 15024927

[B27] Khantikul N Butraporn P Kim HS Leemingsawat S Tempongko MASB Suwonkerd W Adherence to antimalarial drug therapy among vivax malaria patients in Northern Thailand J Health Popul Nutr 2009 27 4 13 19248643 10.3329/jhpn.v27i1.3313 PMC2761802

[B28] Pedro RS Tratamento farmacológico da malária em um instituto de pesquisa clínica no Rio de Janeiro. MSc Thesis 2011 Fundação Oswaldo Cruz, Instituto de Pesquisa Clínica Evandro Chagas, Rio de Janeiro

[B29] Morisky DE Green LW Levine DM Concurrent and predictive validity of a self-reported measure of medication adherence Med Care 1986 24 67 73 10.1097/00005650-198601000-00007 3945130

[B30] Nogueira FHA Moreira-Campos LM Santos RLC Pianetti GA Quality of essential drugs in tropical countries: evaluation of antimalarial drugs in the Brazilian Health System Rev Soc Bras Med Trop 2011 44 582 586 10.1590/S0037-86822011000500010 22031073

[B31] Rodrigues LNC Watanabe SP Ferraz HG In vitro dissolution profile of primaquine tablets available for malaria treatment in Brazil Rev Soc Bras Med Trop 2008 41 41 45 10.1590/S0037-86822008000100008 18368269

[B32] Brasil P Guaraldo L A importância de uma unidade sentinela de vigilância em malária na região extra-amazônica, referência para viajante Boletim Farmacovigilância 2007 4 2

[B33] Galappaththy GNL Omari AAA Tharyan P Primaquine for preventing relapses in people with Plasmodium vivax malaria Cochrane Database of Syst Rev 2007 Issue 1 CD004389 10.1002/14651858.CD004389.pub2 17253504

[B34] Ward SA Mihaly GW Edwards G Looareesuwan S Phillips RE Chanthavanich P Warrell DA Orme MLE Breckenridge AM Pharmacokinetics of primaquine in man. II. Comparison of acute vs chronic dosage in Thai subjects Br J Clin Pharmac 1985 19 751 755 10.1111/j.1365-2125.1985.tb02710.x PMC1463877 4027118

[B35] Mihaly GW Ward SA Edwards G Nicholl DD L’eorme M Breckenridge AM Pharmacokinetics of primaquine in man. I. Studies of the absolute bioavailability and effects of dose size Br J Clin Pharmac 1985 19 745 750 10.1111/j.1365-2125.1985.tb02709.x PMC1463857 4027117

[B36] Muhamad P Ruengweerayut R Chacharoenkul W Rungsihirunrat K Na-Bangchang K Monitoring of clinical efficacy and in vitro sensitivity of Plasmodium vivax to chloroquine in area along Thai Myanmar border during 2009–2010 Malar J 2011 10 44 10.1186/1475-2875-10-44 21324161 PMC3055225

[B37] Wells TNC Burrows JN Baird JK Targeting the hypnozoite reservoir of Plasmodium vivax: the hidden obstacle to malaria elimination Trends Parasitol 2010 26 145 151 10.1016/j.pt.2009.12.005 20133198

[B38] Abdon NP Pinto AYN Silva RSU Souza JM Avaliação da resposta aos esquemas de tratamento reduzidos para malária vivax Rev Soc Bras Med Trop 2001 34 343 348 11562727 10.1590/s0037-86822001000400006

[B39] Schwartz E Regev-Yochay G Kurnik D Short report: a consideration of primaquine dose adjustment for radical cure or Plasmodium vivax malaria AmJTrop Med Hyg 2000 62 393 395 10.4269/ajtmh.2000.62.393 11037784

[B40] Santos JB Luz FCO Deckers FAL Tauil PL Subdoses of primaquine in overweight patients and malaria vivax relapses: report of two cases in the Federal District, Brazil Rev Soc Bras Med Trop 2010 43 749 750 10.1590/S0037-86822010000600033 21181040

[B41] World Health Organization Guidelines for the treatment of malaria 2010 WHO, Geneva 25473692

[B42] Guidelines for Treatment of Malaria in the United States 2001 CDC http://www.cdc.gov/malaria/resources/pdf/treatmenttable.pdf

[B43] Laloo DG Shingadia D Pasvol G Chiodini PL Whitty CJ Beeching NJ Hill DR Warrell DA Bannister BA UK malaria treatment guidelines J Infect 2007 54 111 121 10.1016/j.jinf.2006.12.003 17215045

[B44] Centre for Disease Control Malaria guidelines for health professionals in the northern territory 2007 5 CDC, Australia

[B45] Fernando D Rodrigo C Rajapakse S Primaquine in vivax malaria: an update and review on management issues Malar J 2011 10 351 10.1186/1475-2875-10-351 22152065 PMC3306765

